# Microfluidic Device to Measure the Speed of *C. elegans* Using the Resistance Change of the Flexible Electrode

**DOI:** 10.3390/mi7030050

**Published:** 2016-03-19

**Authors:** Jaehoon Jung, Masahiro Nakajima, Masaru Takeuchi, Zoran Najdovski, Qiang Huang, Toshio Fukuda

**Affiliations:** 1 Department of Micro-Nano Systems Engineering, Nagoya University, Furo-cho, Chikusa-ku, Nagoya 464-8601, Japan; takeuchi@mein.nagoya-u.ac.jp; 2 Center for Micro-Nano Mechatronics, Nagoya University, Furo-cho, Chikusa-ku, Nagoya 464-8601, Japan; nakajima@mein.nagoya-u.ac.jp; 3 Center for Intelligent Systems Research, Deakin University, Waurn Ponds, Geelong 3216, Australia; zoran.najdovski@deakin.edu.au; 4 Institute for Advanced Research, Nagoya University, Furo-cho, Chikusa-ku, Nagoya 464-8601, Japan; tofukuda@nifty.com; 5 Department of Mechatronics Engineering, Meijo University, Shiogamaguchi, Tenpa-ku, Nagoya 468-0073, Japan; 6 Intelligent Robotics Institute, School of Mechatronic Engineering, Beijing Institute of Technology, 5 South Zhongguancun Street, Beijing 100081, China; qhuang@bit.edu.cn; 7 Medical Device Development Center, Daegu-Gyeongbuk Medical Innovation Foundation (DGMIF), 80 Cheombok-Ro, Dong-gu, Daegu 41061, Korea

**Keywords:** microfluidic device, *C. elegans*, flexible electrode

## Abstract

This work presents a novel method to assess the condition of *Caenorhabditis elegans* (*C. elegans*) through a resistance measurement of its undulatory locomotion speed inside a micro channel. As the worm moves over the electrode inside the micro channel, the length of the electrode changes, consequently behaving like a strain gauge. In this paper, the electrotaxis was applied for controlling the direction of motion of *C. elegans* as an external stimulus, resulting in the worm moving towards the cathode of the circuit. To confirm the proposed measurement method, a microfluidic device was developed that employs a sinusoidal channel and a thin polydimethylsiloxane (PDMS) layer with an electrode. The PDMS layer maintains a porous structure to enable the flexibility of the electrode. In this study, 6 measurements were performed to obtain the speed of an early adult stage *C. elegans*, where the measured average speed was 0.35 (±0.05) mm/s. The results of this work demonstrate the application of our method to measure the speed of *C. elegans* undulatory locomotion. This novel approach can be applied to make such measurements without an imaging system, and more importantly, allows directly to detect the locomotion of *C. elegans* using an electrical signal (*i.e.*, the change in resistance).

## 1. Introduction

The nematode *Caenorhabditis elegans* (*C. elegans*) is considered a model organism and a bio indicator due to its many advantages, including its microscopic size, short life span and fast generation time, transparent body, well known cell lineage and genome map, and relevance to human diseases [1,2,3,4,5,6,7,8,9,10]. Furthermore, an external stimuli such as chemical, toxin or drug allows for the detection of *C. elegans*’ condition. Features such as its body size, locomotion and life span have demonstrated correlation to such stimuli [11,12,13,14,15,16].

The locomotion of *C. elegans* is a feature that can ascertain its condition initiated by an external stimulus. This locomotion is a natural behavior that enables *C. elegans* to move to favorable surroundings (e.g., a location of food), or to escape from harmful and noxious stimuli [16,17,18,19]. When *C. elegans* moves on the surface, the worm’s movement exhibits an undulatory crawling motion (*i.e.*, sinusoidal pattern) produced by a wave of muscular contraction and relaxation as it moves along the body. Typically, there are four kinds of motion: forward crawling, backward crawling, omega turn, and resting [18,19,20,21,22]. The generation of locomotion begins by sensing its environment, such as chemotaxis, thermotaxis, and aerotaxis. If *C. elegans* senses an external stimulus within its environment using its sensory neuron, the stimulus generates a signal on a cell-level, which in turn controls a motor neuron to move its muscles, and subsequently create the described locomotion [8,20,21,22,23,24]. Therefore, the locomotion of *C. elegans* has useful information regarding the functionality of the neuronal and muscular system of the worm. As a result, the locomotion of *C. elegans* has a correlation with an external stimulus such as a heavy metal and neurotoxin, and is therefore considered to be a key-factor in determining the worm’s condition. For these reasons, research has focused on characterizing the locomotion of *C. elegans* [25,26,27]. The use of microfluidic devices for the study of *C. elegans* locomotion has successfully investigated its locomotion, and additionally, its force level [24,28,29], adaptability [30], and behavior [19]. In these works, the use of an imaging system and software to observe the undulatory locomotion of *C. elegans* limited the size of the observing system, therefore, making it difficult to develop a small and portable biological assay for *C. elegans*. 

In this study, we propose a novel method, through the use of a microfluidic device and an optical microscope, to evaluate the locomotion of *C. elegans* in real time without an imaging system. Using our method, we are able to observe the locomotion of *C. elegans*. The speed of *C. elegans* was measured in addition to the locomotion of the worm. To measure the speed of *C. elegans*, the microfluidic device must encompass two features: (1) it can control the motion direction of the worm without force (e.g., applying pressure)—the worm must move on its own accord; and (2) it can directly convert the motion of *C. elegans* into an electrical signal. Firstly, motion direction control is achieved through electrotaxis. Electrotaxis is the ability of *C. elegans* to respond to an electrical signal. It may have evolved as a host finding cue in parasitic nematodes [31,32,33]. Rezai and colleagues succeeded in manipulating the motion direction of *C. elegans* in a microfluidic channel using electrotaxis for the first time [34]. In this work, although *C. elegans* experienced different electrotaxis outcomes depending on the larval stage, they all moved toward a cathode in a voltage electric field. This suggests that electrotaxis can be utilized to manipulate the motion direction of *C. elegans*. Second, the conversion from the motion of *C. elegans* to an electrical signal was achieved by the development of a flexible electrode. The concept of a flexible electrode is that it behaves the same as a strain gauge. If the length of the electrode changes by the motion of *C. elegans*, this creates a change in resistance of the flexible electrode, which correlates to the locomotion of *C. elegans*. Using our proposed device, the motion of *C. elegans* was detected by a flexible electrode and the speed of the worm was obtained. These results demonstrate the capability of the proposed microfluidic device to study the locomotion of *C. elegans* without an imaging system, and by a simple experimental setup for real-time evaluation.

## 2. Materials and Methods

### 2.1. Microfluidic Device Design

**Figure 1:**
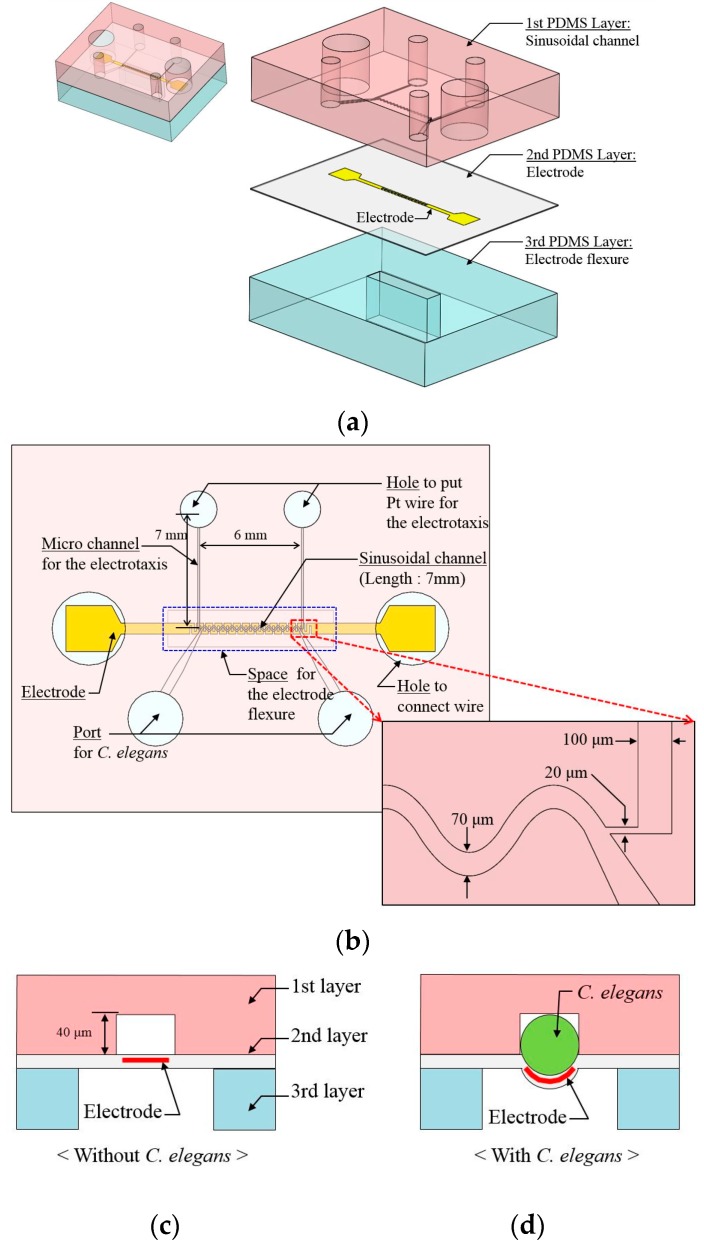
(**a**) Schematic diagrams of our proposed microfluidic device to measure the speed of *C. elegans*. It is composed of three polydimethylsiloxane (PDMS) layers and each PDMS layer had its own function. (**b**) Top view of our proposed microfluidic device to measure the speed of *C. elegans*. A micro channel for the electrotaxis is connected to a sinusoidal channel. The sinusoidal channel is on the electrode. (**c**) Cross sectional view of our prosed device. (**d**) When *C. elegans* is loaded into the sinusoidal channel, the worm can be introduced into the channel by the expansion of the second PDMS layer, which deforms the flexible electrode.

The proposed microfluidic device is composed of three polydimethylsiloxane (PDMS; SILPOT 184, Dow corning Toray Co., Tokyo, Japan) layers (Figure 1a). The first PDMS layer has a pattern to guide the motion of *C. elegans*, including a sinusoidal channel and the micro channel for electrotaxis (Figure 1b).

The sinusoidal channel is a channel to guide the undulatory motion of *C. elegans*. While the wave length and the amplitude of the undulatory motion is different for the various larval stages and types of *C. elegans* (*i.e.*, mutant), the presented sinusoidal channel is suitable for the motion of *C. elegans* [18,19,20,21,22]. In this study, the sinusoidal channel has a width of 70 μm, a wavelength of 500 μm, and amplitude of 100 μm. It was designed for an adult *C. elegans* motion [19]. The height of the sinusoidal channel is approximately 40 μm, due to being designed for an early adult stage (diameter ≈ 50 μm). For tight connection between a flexible electrode and the worm, the height of the sinusoidal channel is designed to be 40 μm (Figure 1c). When the *C. elegans* is loaded into the channel, it can be introduced into a channel by the expansion of the second PDMS layer, by way of the flexible electrode deforming (Figure 1d). The micro channel is connected to the sinusoidal channel for the electrotaxis of *C. elegans*. These two channels (*i.e*., a sinusoidal channel and micro channel for electrotaxis) were connected with a narrow channel 20 μm in width. This prevented the worm from being introduced into the micro channel for electrotaxis (Figure 1b).

**Figure 2:**
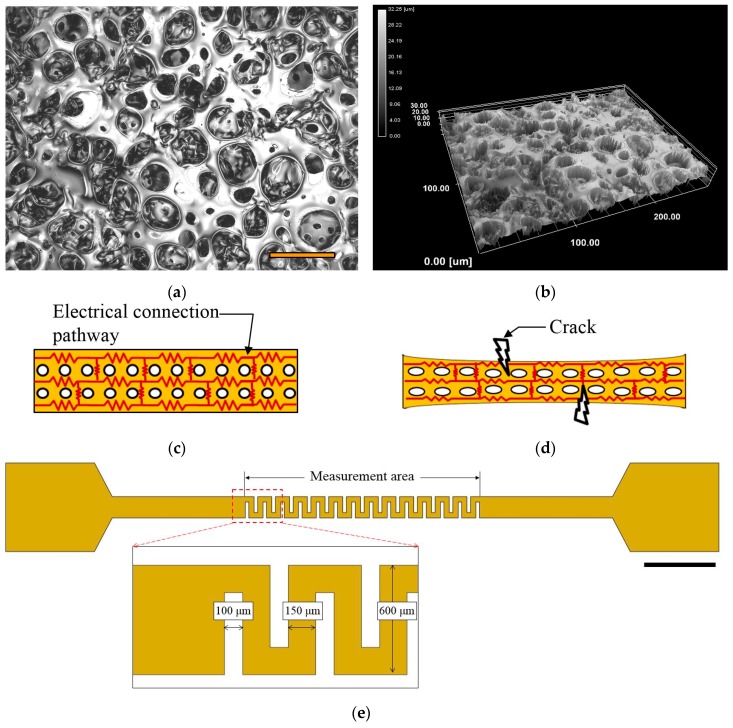
(**a**) Laser microscopic image of the second PDMS layer. Scale bar marks 50 μm. (**b**) 3D scanning image. The average depth of pores is approximately 17 μm. (**c**) Initial state of an electrode. The porous PDMS layer had a lot of electrical pathways on the surface. (**d**) Though some electrical connections were broken by the external force (e.g., the extension force in fabrication procedures), the electrode could maintain the electrical connection. (**e**) The electrode design on the second PDMS layer. The red rectangle showed the size of electrode. Scale bar marks 2 mm.

The second PDMS layer is a thin PDMS layer with a thickness of approximately 50 μm. It has a porous structure on the surface. The average depth of the pores is approximately 17 μm (Figure 2a,b). The large number of pores on the surface of the PDMS layer produced a sponge-like mesh structure. The mesh created the electrical connection pathways on the surface of the electrode (Figure 2c). For these reasons, even though some electrical connections were broken by the external force (e.g., the extension force in fabrication procedures), the electrode could maintain the electrical connection (Figure 2d), [35]. The electrode is fabricated by Cr and Au sputtering. The electrode was used to detect the motion of *C. elegans* by the change in resistance, which acted like a strain gauge. The electrode is 150 μm in width and the gap between electrodes is 100 μm. To cover the area of the sinusoidal channel, the amplitude of the electrode is 600 μm (Figure 2e). The third PDMS layer has a space for flexure of the electrode. These three PDMS layers are bonded by O_2_ plasma treatment.

### 2.2. Fabrication of the Microfluidic Device

**Figure 3:**
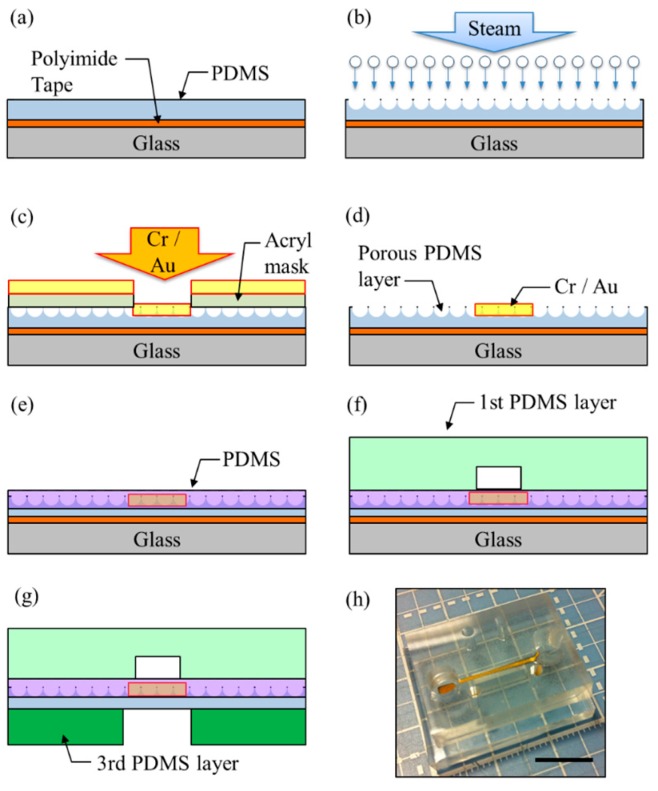
Fabrication procedures: (**a**) PDMS coated by spin coating. (**b**) Uncured PDMS was placed in an autoclave machine for steam etching. (**c**) An acryl mask was used to make a pattern for the electrode. Cr (10 min) and Au (10 min) were sputtered in order. (**d**) After removing the acryl mask, Cr/Au electrode remained on the porous PDMS layer. (**e**) PDMS was coated on the porous PDMS layer to make a surface smooth and protect an electrode. (**f**) The first PDMS layer was bonded on the second PDMS layer to fasten the electrode on the porous PDMS. (**g**) The third PDMS layer was bonded under the second PDMS layer. (**h**) Fabricated microfluidic device. Scale bar marks 1 cm.

Each PDMS layer was fabricated via its own method. The first PDMS layer was fabricated by soft-lithography [36,37]. The second PDMS layer was fabricated by steam etching [35], and the third PDMS layer was fabricated using aluminum mold to create space for flexure of the electrode. Figure 3 shows the fabrication procedures of the microfluidic device.

The microfluidic device was fabricated from the second PDMS layer and is a flexible electrode, same as below. PDMS was coated on the glass by spin coating. The glass was covered by a polyimide. The polyimide’s role was to easily detach the PDMS membrane from the glass (Figure 3a). Uncured PDMS was put in an autoclave machine for steam etching for 10 min (Figure 3b). During steam etching, the porous structure was created due to the following: Initially, the steam heats the uncured PDMS layer surface and creates holes. Next, the air inside the PDMS burst by the heating process [35].

**Figure 4:**
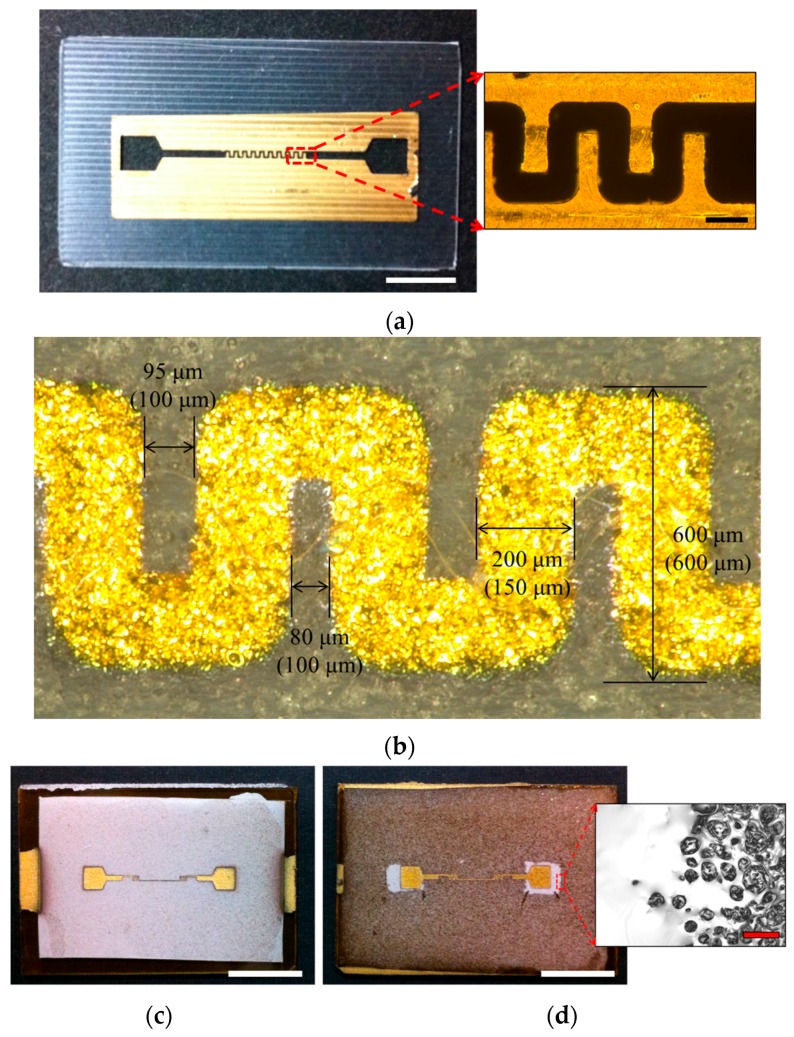
Photograph of the electrode: (**a**) Photograph and microscope image of an acryl mask. Each scale bar marks 5 mm (white line) and 200 μm (black line). (**b**) Photograph of the electrode. Though the size of the electrode changed due to the processing error of the mask, it maintained the pattern. Figures in parenthesis refer to the designed size. (**c**) The porous PDMS layer before coating PDMS. (**d**) The porous PDMS layer after PDMS coating. The laser microscopic image showed the boundary between coated and uncoated area. Scale bars mark 1 cm (black line) and 50 μm (red line).

An acryl mask was used to make a pattern for the electrode during sputtering (Figure 4a). The thickness of the acryl mask was 200 μm and the shape of the electrode was the same design (Figure 2e). Cr (10 min) and Au (10 min) were sputtered in order (Figure 3c). After removing the acryl mask, the electrode remained on the porous PDMS (Figure 3d). Though the size of the electrode changed due to the processing error of the mask, it maintained the pattern (Figure 4b).

To make a smooth surface and protect the electrode, PDMS was coated on the porous PDMS layer one more time (Figure 3e). After coating PDMS, the color of the porous PDMS layer changed because the pores on the surface were covered by PDMS. From the laser microscopic image, the difference between coated and uncoated area was confirmed (Figure 4c,d). By coating PDMS on the porous PDMS layer, the electrode was protected from the motion of *C. elegans*, and decreased the damaging effects on the porous PDMS layer (the coated area was smooth).

Before removing the second PDMS layer from the glass, the first PDMS layer was bonded to the second PDMS layer by the O_2_ plasma method to secure the electrode on the porous PDMS (Figure 3f). The third PDMS layer was bonded under the second PDMS layer by O_2_ plasma method (Figure 3g). Figure 3h shows the fabricated microfluidic device.

### 2.3. Preparation of C. elegans Strain

The nematode *C. elegans* (N2 Bristol) was used in this experiment. *C. elegans* was grown on a nematode-growth-medium (NGM) agar plate (3 g NaCl, 17 g agar, 2.5 g peptone, 975 mL H_2_O, 1 mL CaCl_2_ (1M), 1 mL MgSO_4_ (1M), 25 mL KPO_4_ buffer (pH 6.0), 1 mL cholesterol in ethanol (5 mg·mL^−1^) [38]) seeded with an OP50 strain of *Escherichia coli* (*E. coli*) and maintained at 15 °C so that it would slowly develop [39]. For the synchronization of the age of *C. elegans*, eggs were isolated from gravid *C. elegans* using a bleaching mixture (4%–6% sodium hypochlorite (NaClO), 5 M KOH [40]). They were kept on a plate with K-medium (53 mM NaCl, 32 mM KCl [41]) and *E. coli* (OP50) at 22 °C to hatch, and they grew to an early adult stage over 55 h [11].

## 3. Results and Discussions

### 3.1. Electrotaxis Test Result

**Figure 5:**
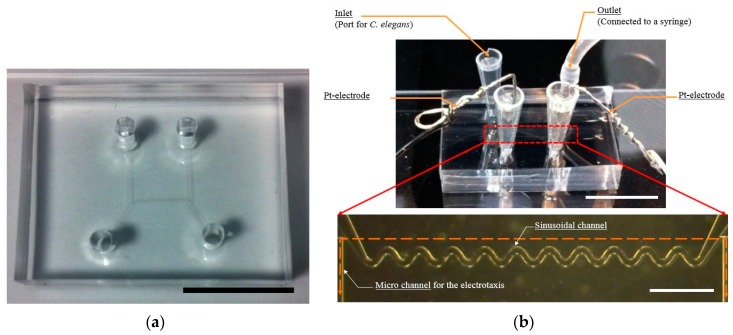
(**a**) Photograph of the microfluidic device for the electrotaxis test. Scale bar marks 1 cm. (**b**) Photograph of the experimental setup and a microscopic image of the sinusoidal channel. Electric current flowed following the micro channel (orange line). Scale bars mark 1 cm (top) and 1 mm (bottom).

When a direct electric current (DC) was applied to K-medium, bubbles were generated at the electrodes by electrolysis. Bubbles had a detrimental effect on electrotaxis, as they can create a gap between the electrode and the K-medium. To solve this problem, and generate an effective electrical field in a sinusoidal channel, a micro channel was added at the end of the sinusoidal channel (Figure 1b). For the electrotaxis experiment, the microfluidic device was fabricated (Figure 5a). It had a micro channel and a sinusoidal channel. The structure was the same as the first PDMS layer (Figure 1b). Figure 5b shows the experimental setup. Pt electrodes were connected to each hole. As a result, electric current flowed following micro channels (*i.e.*, a sinusoidal channel and micro channel for electrotaxis, the orange line in Figure 5b). The micro channels worked as an electrical pathway that had electrical resistance; therefore, they could make an electrical field without bubbles. (see Supplementary Material Figure S1).

**Figure 6:**
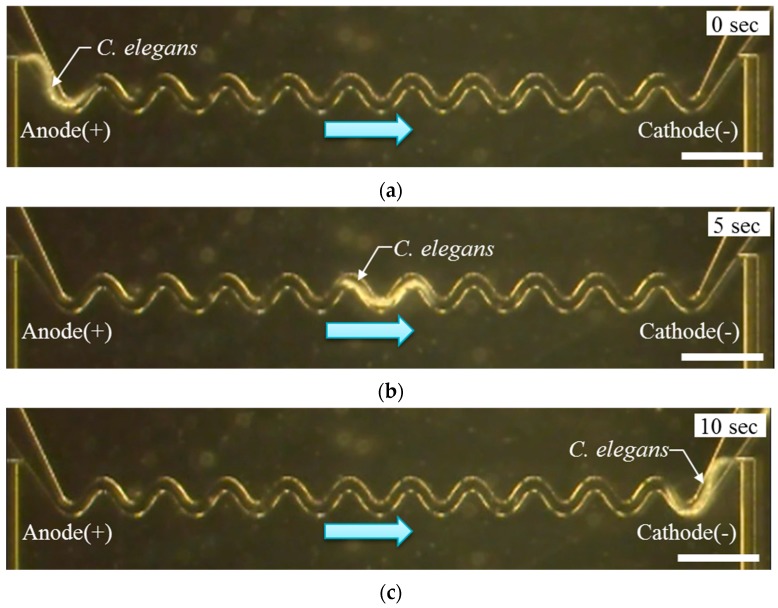
The measurement result of the electrical current using the micro channel. (**a**–**c**) Experiment result of electrotaxis over time: (a) 0 s; (b) 5 s; and (c) 10 s after applying 5 V (~2.4 V/cm). Scale bar marks 1 mm.

In order to load *C. elegans* into the sinusoidal channel, the worm was introduced to the inlet of the sinusoidal channel by a syringe (*i.e.*, negative pressure) (Figure 5b). Following this stage, the negative pressure was stopped. The negative pressure was a requirement for introducing the worm into the system. To confirm the electrotaxis of *C. elegans*, a direct current (DC) voltage was applied to *C. elegans* from 1 to 10 V using a regulated DC power supply (PMC 250-0.25A, Kikusui electronics Co., Yokohama, Japan) [35]. When *C. elegans* was exposed to approximately 5 V (~2.4 V/cm), the worm demonstrated the effect of electrotaxis (Figure 6a–c). When the position of the cathode was changed, the worm moved to the cathode. When *C. elegans* was exposed to a higher voltage of 9 V (~4.3 V/cm), occasionally the worm did not display the effect of electrotaxis. In addition, the worm stopped within the sinusoidal channel and appeared to be paralyzed. From this experiment, we were able to confirm the method of controlling the motion of *C. elegans* through electrotaxis.

### 3.2. Speed Measurement Using Resistance Change

**Figure 7:**
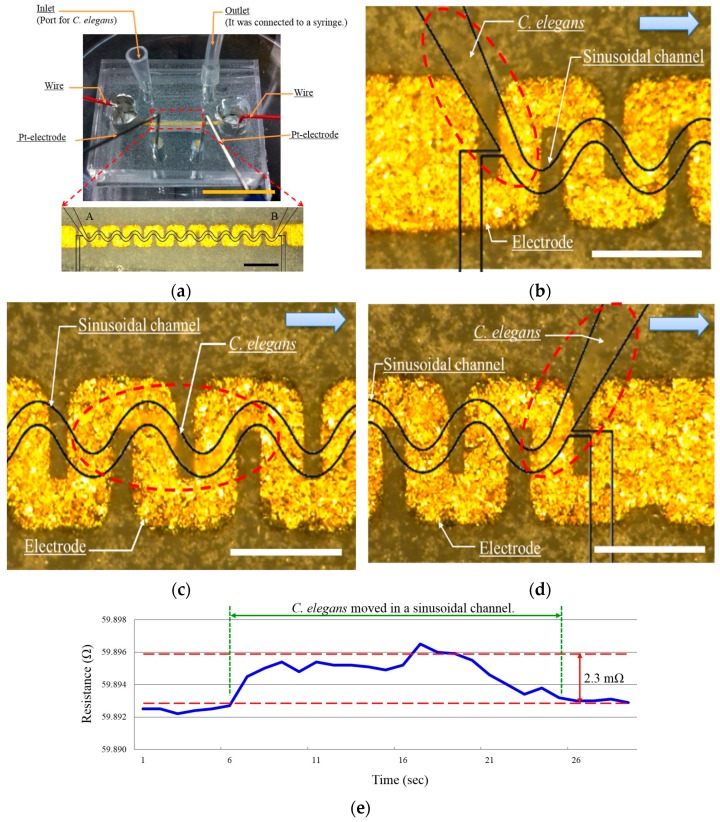
Experimental result of speed measurement. (**a**) Fabricated microfluidic device for the speed measurement. The microfluidic device was translucent, therefore the guide line (Black line) was added to show the position of the sinusoidal channel. Wires were connected by Ag paste. Scale bars mark 1 cm (top) and 1 mm (bottom). (**b**–**d**) motion of *C. elegans*: (b) 0 s; (c) 6 ; and (d) 22 s after applying 5 V (~2.4 V/cm). The microfluidic device was translucent, therefore it could carefully observe *C. elegans* in the sinusoidal channel. Scale bar marks 500 μm. (**e**) Speed measurement through the resistance change. The resistance was changed while *C. elegans* moved on the electrode. From the resistance change, the travel time was obtained.

Figure 7a shows a fabricated microfluidic device and the experimental setup. At the end of the electrode, a wire was connected by Ag paste. An LCR meter (ZM 2371, NF Co., Yokohama, Japan) was used to measure resistance and to apply an AC voltage (1 V, 1 kHz) to the device. Similar to the electrotaxis test, a DC voltage of 5 V was applied by a regulated DC power supply (MC 250-0.25A, Kikusui electronics Co.).

To measure the speed of *C. elegans* undulatory motion, an adult *C. elegans* was loaded into the inlet of the sinusoidal channel, utilizing the same method used in the electrotaxis experiment above. The worm was introduced to the inlet of the sinusoidal channel with a syringe, then the negative pressure was released to prevent the pressure effect on the electrode. This process deformed the flexible electrode, and therefore caused a change in resistance in our proposed microfluidic device. Next, the motion of *C. elegans* was controlled by electrotaxis. As shown in Figure 7a, during the fabrication method, the color of the second PDMS layer changed (Figure 4d). Therefore, it was challenging to observe *C. elegans* within the sinusoidal channel. Depending on the position of the cathode, *C. elegans* moved from one end point to the other (e.g., from “A” to “B” or from “B” to “A”) (Figure 7b–d) (See the Movie S1). When *C. elegans* moved within the sinusoidal channel from one end point to the other, for example A→B (as shown in Figure 7a), there was a change in the measured resistance (as shown the graph in Figure 7e). From this change in resistance, the travel time of *C. elegans* was measured six times. Measurement results are presented in Table 1. The results show the travel time was 20.3 (±3.0) s, and the change in resistance was 1.8 (±0.6) mΩ. From the travel time, the average speed obtained was 0.35 (±0.05) mm/s. 

These outcomes confirm the capability of our presented method to evaluate the locomotion of *C. elegans* by measuring its average speed through the change in resistance. This is achieved without the use of an imaging system.

## 4. Conclusions

In this study, a new method is proposed to evaluate the locomotion of *C. elegans* without an imaging system. Since the flexible electrode was incorporated with our proposed microfluidic device, we were able to measure the average speed of *C. elegans* by the change in resistance. During the speed measurement, electrotaxis of *C. elegans* was used to control the motion direction of the worm without a forced method (e.g., applying pressure). The worm must move on its own accord. With our proposed microfluidic device, the average speed measurement was conducted using the change in resistance of the flexible electrode. While the *C. elegans* moved in the sinusoidal channel, the resistance of the flexible electrode changed. 

In this work, we have confirmed the application of our method and apparatus to measure the average speed of *C. elegans* by the change in resistance. This is a novel method to directly convert the locomotion of *C. elegans* into an electrical signal (*i.e.*, a change in resistance). As a result, it is applied to study the nematode. The nematode has the same locomotion pattern as *C. elegans* such as *Oesophagostomum* species parasites of humans [29]. Furthermore, a basic principle of our method is the same as a strain gauge, therefore it can be used as a sensor to detect the environment in a microfluidic device such as pressure in a micro channel. This will serve as a stepping stone for the development of a portable nematode observation systems to detect the condition of the worm without an imaging system.

## Figures and Tables

**Table 1:** Measurement result of the travel time and change in resistance. SD: Standard deviation.

No	Travel Time (s)	Resistance Change (mΩ)	Speed (mm/s)
1	18.0	1.7	0.39
2	20.0	1.4	0.35
3	23.0	2.0	0.30
4	25.0	1.0	0.28
5	18.0	2.5	0.39
6	18.0	2.3	0.39
Average (±SD)	20.3 (±3.0)	1.8 (±0.6)	0.35 (±0.05)

## Supplementary Materials

The following are available online at http://www.mdpi.com/2072-666X/7/3/50/s1, Figure S1: Experimental result of the electrotaxis test: (a) The measurement result of the electrical current. The electric current was smaller in the micro channel than in the petri dish. Bubbles formed at approximately 450 μA in the petri dish. The green arrow indicates where bubbles formed. (b) The measurement result of the electrical current using a micro channel. Video S1: The motion of *C. elegans* during the speed measurement.
