# Supplementary material for: Microfluidic Device to Measure the Speed of C. elegans Using the Resistance Change of the Flexible Electrode
Source: Micromachines (Basel). 2016 Mar 19;7(3):50. doi: 10.3390/mi7030050 (PMC6190434; doi:10.3390/mi7030050)
Supplement: Supplementary file 1 [file micromachines-07-00050-s001.zip › supplymentary materials.pdf]

Supplementary Materials: Microfluidic Device to Measure the Speed of *C. elegans* using the Resistance Change of the Flexible Electrode

Jaehoon Jung, Masahiro Nakajima, Masaru Takeuchi, Zoran Najdovski, Qiang Huang and Toshio Fukuda

The Role of A Micro Channels on the Electrotaxis

To compare the electric condition (e.g., current and resistance), depending on the experimental condition of whether to use a micro channel or not, the electrical current was measured. The distance between electrodes was the same in both conditions to make the same electric field (V/cm). A petri dish was used for the case without a micro channel. Bubble formed at approximately 450 μA in the petri dish, represented by the green line in Figure S1a. The electric current was smaller in the micro channel than in the petri dish. When the micro channel was used for electrotaxis, it worked as an electrical pathway, similar to a wire. Because electrical resistance was bigger in the micro channel than in the petri dish, the micro channel prevented electrical current from increasing in comparison to the petri dish. Therefore, the micro channel kept an electrical field without bubble.

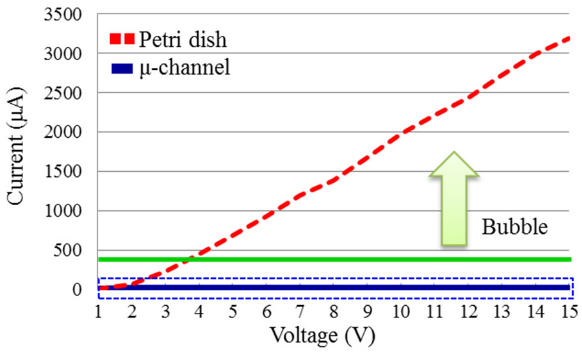

(**a**)

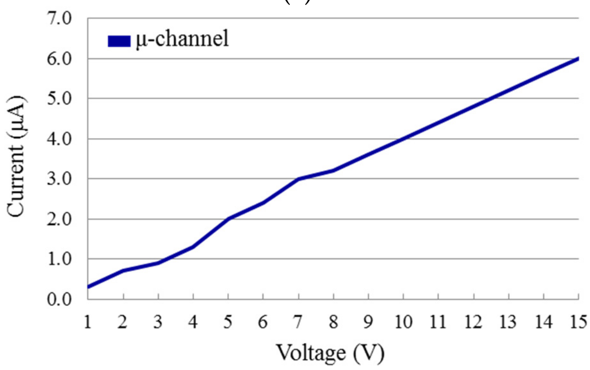

(**b**)

Figure S1. Experimental result of the electrotaxis test: (**a**) The measurement result of the electrical current. The electric current was smaller in the micro channel than in the petri dish. Bubbles formed at approximately 450 μA in the petri dish. The green arrow indicates where bubbles formed. (**b**) The measurement result of the electrical current using a micro channel.
